# Exploring the association between gastroesophageal reflux disease and hyperlipidemia through bioinformatics and Mendelian randomization

**DOI:** 10.1097/MD.0000000000050767

**Published:** 2026-09-18

**Authors:** Yongming Sun, Wenyan Wang, Xiaodong Xing, Wodele Kang, Dawei Cheng, Jingbo Zhai

**Affiliations:** a Department of Operating Room, Affiliated Hospital of Inner Mongolia Minzu University, Tongliao, China; b College of Medicine, Inner Mongolia Minzu University, Tongliao, China; c Department of Infectious Diseases, Beidahuang Industry Group General Hospital, Harbin, China; d College of Medicine, Inner Mongolia Minzu University/Key Laboratory of Zoonose Prevention and Control at Universities of Inner Mongolia Autonomous Region, Tongliao, China; e College of Medicine, Inner Mongolia Minzu University/Brucellosis Prevention and Treatment Engineering Research Center of Inner Mongolia Autonomous Region, Tongliao, China.

**Keywords:** bi-disease analysis, bioinformatic, gastroesophageal reflux disease, hyperlipidemia, Mendelian randomization

## Abstract

Observational research has found a relationship between gastroesophageal reflux disease (GERD) and hyperlipidemia (HLP). This study explored the relationship between GERD and HLP using bioinformatics and Mendelian randomization (MR) methods. We first conducted bioinformatics analyses to identify differentially expressed genes (DEGs) from disease datasets and then performed a two-sample MR analysis with aggregated genome-wide association study (GWAS) data to investigate the relationship. 11 DEGs were shared between GERD and HLP. According to the Kyoto Encyclopedia of Genes and Genomes (KEGG) enrichment analysis, cholesterol metabolism, renin secretion, and glycerolipid metabolism may represent common underlying mechanisms between the 2 diseases. The Inverse Variance Weighted (IVW) method identified *P* values of < .001 and .003 between GERD and HLP in the primary and replication MR analysis, respectively. The results were robust, as confirmed with the heterogeneity and pleiotropy tests. This study revealed the causal relationship between GERD and HLP through comprehensive approaches. The implicated shared genes and pathways offer valuable insights into potential therapeutic avenues and highlight the importance of addressing this link in clinical practice.

## 1. Introduction

Gastroesophageal reflux disease (GERD) is a disorder of the digestive tract caused by the backward flow of stomach acids, food, and fluids into the esophagus. This reflux may irritate the esophagus lining, leading to symptoms like heartburn, chest pain, and difficulty swallowing. This heartburn can extend to the back, particularly following meals or in a reclined posture, and it can often be relieved through the application of antacids.^[1]^ GERD prevalence is 10–20% in Western countries and 5% in Asian regions.^[2]^ GERD may result in complications such as esophagitis, Barrett’s esophagus, and a heightened risk of esophageal cancer. Further complications include chronic obstructive pulmonary disease, angina, and symptoms and conditions of the laryngeal and upper respiratory tract.^[3]^ Management approaches for GERD include lifestyle changes, pharmaceutical interventions, and surgical procedures. Lifestyle adaptations encompass steering clear of foods that relax the lower esophageal sphincter and pursuing weight loss. Common medications include proton-pump inhibitors and histamine H2 receptor antagonists. The surgical treatment typically involves the Nissen fundoplication.^[4]^

Hyperlipidaemia (HLP) is a prevalent metabolic condition marked by high levels of cholesterol and triglycerides. Clinical subtypes include hypercholesterolemia, hypertriglyceridemia, and mixed hyperlipidemia.^[5]^ HLP branches into primary and secondary categories based on causative factors. Primary HLP results from single or multiple gene defects and is also referred to as familial HLP. Secondary HLP, also known as acquired HLP, is associated with conditions including diabetes, nephrotic syndrome, hypothyroidism, and the administration of medications like beta-blockers and corticosteroids.^[6]^ Modifiable factors that influence HLP include a diet rich in saturated or trans fatty acids, smoking, insufficient physical activity, and obesity.^[7]^ HLP significantly contributes to the development of atherosclerosis, ultimately leading to ischemic stroke, coronary heart disease, etc.^[8]^ Management strategies for HLP include modifiable lifestyle factors like adopting a healthy diet, weight loss, smoking cessation, and regular exercise. Statins constitute the prevailing class of lipid-lowering medications, accompanied by other agents such as bile acid sequestrants, omega-3 fatty acids, fibrates,^[9]^ and herbal remedies.^[10]^ Despite their role as front-line lipid-lowering agents, statins are associated with potential side effects such as muscle discomfort, acute liver dysfunction, neurological issues, and potentially elevated cancer risk.^[11]^

Although some studies have suggested an association between GERD and cardiovascular conditions such as myocardial infarction, atrial fibrillation, and stroke, as well as established risk factors including body mass index (BMI), high-density lipoprotein (HDL) cholesterol, and triglycerides (TG), a direct causal link between GERD and HLP remains unproven.^[12]^ Recent cross-disease genomic studies have highlighted the genetic overlaps between GERD and other systemic conditions, offering a compelling rationale for using systematic approaches to explore novel disease associations.^[13]^ By utilizing instrumental variables (IVs), such as single nucleotide polymorphisms (SNPs), Mendelian randomization (MR) provides a way of evaluating the causal relationship between risk factors and complex diseases.^[14]^ These SNPs are randomly allocated during conception, thus minimizing the susceptibility to disease-related influences. MR aids in establishing the relationship between exposure and outcome by mitigating the effects of confounding factors and reverse causation.

This study amalgamates bioinformatic methods to dissect GERD and HLP, revealing potential shared genes and underlying mechanisms. Subsequently, a two-sample MR analysis explored the interplay between these 2 conditions and deciphered their clinical significance in the context of management strategies.

## 2. Materials and methods

### 2.1. Data source for joint analysis of GERD and HLP

**Figure 1.**
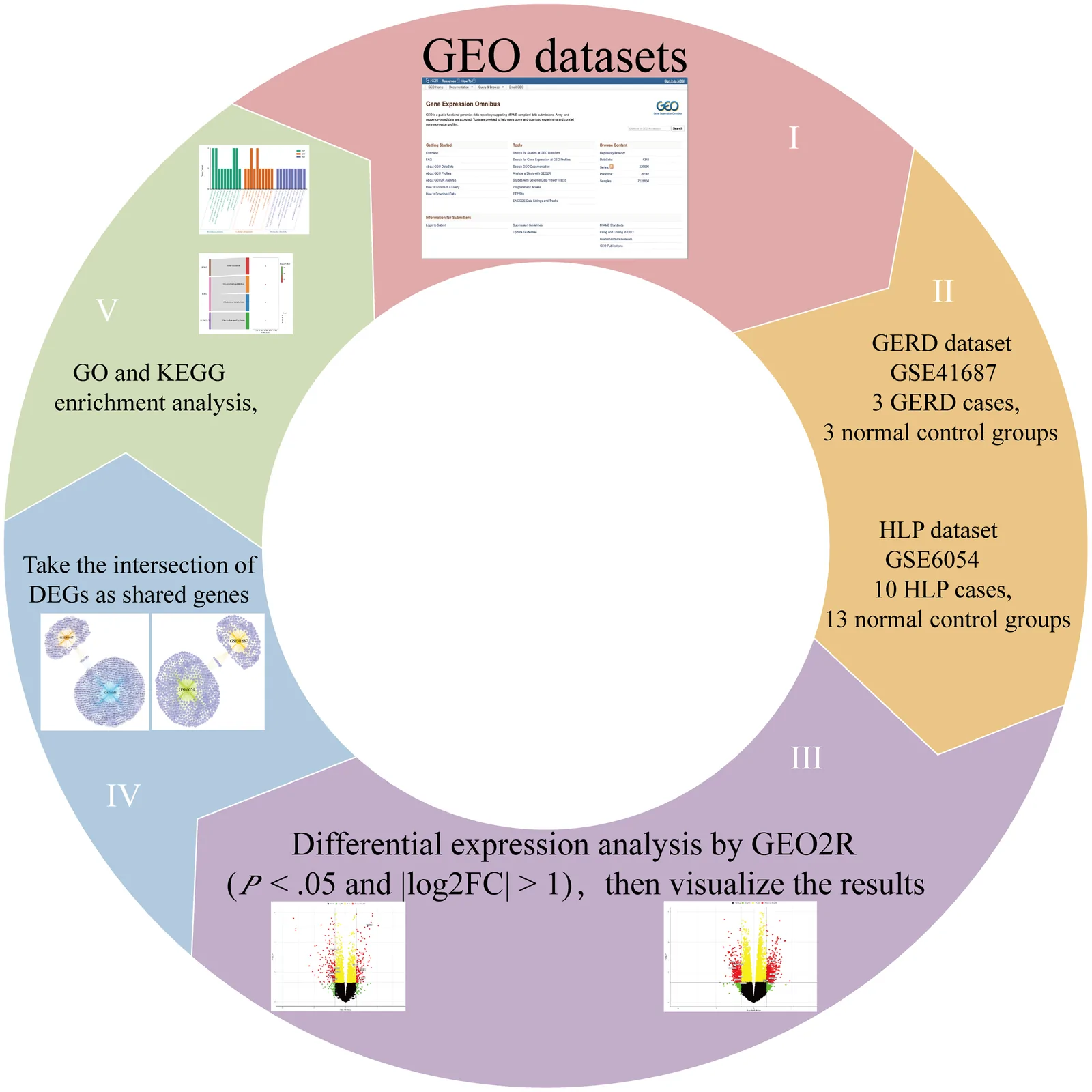
Flowchart of the bioinformatics section. GERD = gastroesophageal reflux disease, HLP = hyperlipidemia. Figure 1. illustrates the overall design and analytical sequence of our bioinformatics approach, enabling readers to quickly grasp the logical flow and the interconnections among the various analytical steps.

The Gene Expression Omnibus (GEO) serves as an open database offering a repository of bioinformatics data, encompassing microarray and advanced sequencing data. This platform incorporates large-scale genomic profiling data obtained from high-throughput platforms. Datasets for GERD and HLP were obtained from GSE41687 (Illumina Genome Analyzer II GPL9115 platform) and GSE6054 (Affymetrix Human Genome U133 Plus 2.0 Array 570 platform), respectively. Within the GSE41687 dataset, the samples include proximal and distal esophageal biopsies from three GERD cases and three normal control groups. The GSE6054 dataset contains monocyte samples from 10 HLP cases and 13 normal control groups. The bioinformatics section flowchart is shown in Figure 1.

### 2.2. Differential analysis

This study employed the online tool GEO2R within the GEO database to conduct differential analysis. GEO2R facilitates differential gene expression analysis and visualization using R language. Our standards for identifying differentially expressed genes (DEGs) were set at *P* < .05 and |log2FC| > 1. Here, FC denotes the fold change in gene expression. Genes with log2FC < 0 denote downregulated genes in comparison to the control group, whereas genes with log2FC > 0 denote upregulated genes. The shared DEGs for GERD and HLP were identified by intersecting up- and down-expressed DEGs derived from the GSE41687 and GSE6054 datasets, respectively.

### 2.3. Function and pathway enrichment analyses

Gene Ontology (GO) and Kyoto Encyclopedia of Genes and Genomes (KEGG) enrichment analyses were conducted using the R package “clusterProfiler,” with a significance threshold of *P* < .05. GO encapsulates three facets of gene function: Biological process (larger processes or “biological programs” constituted by multiple molecular activities, BP), Cellular component (functions of gene products concerning cellular structures and locations, CC), and Molecular function (functions carried out by gene products at the molecular level, MF).^[15]^ KEGG aids in probing the potential pathways that are associated with these genes. Based on the results of the aforementioned bioinformatics section, this study engaged in a two-sample MR analysis for an in-depth exploration of the association between GERD and HLP.

### 2.4. MR analysis data sources

**Figure 2.**
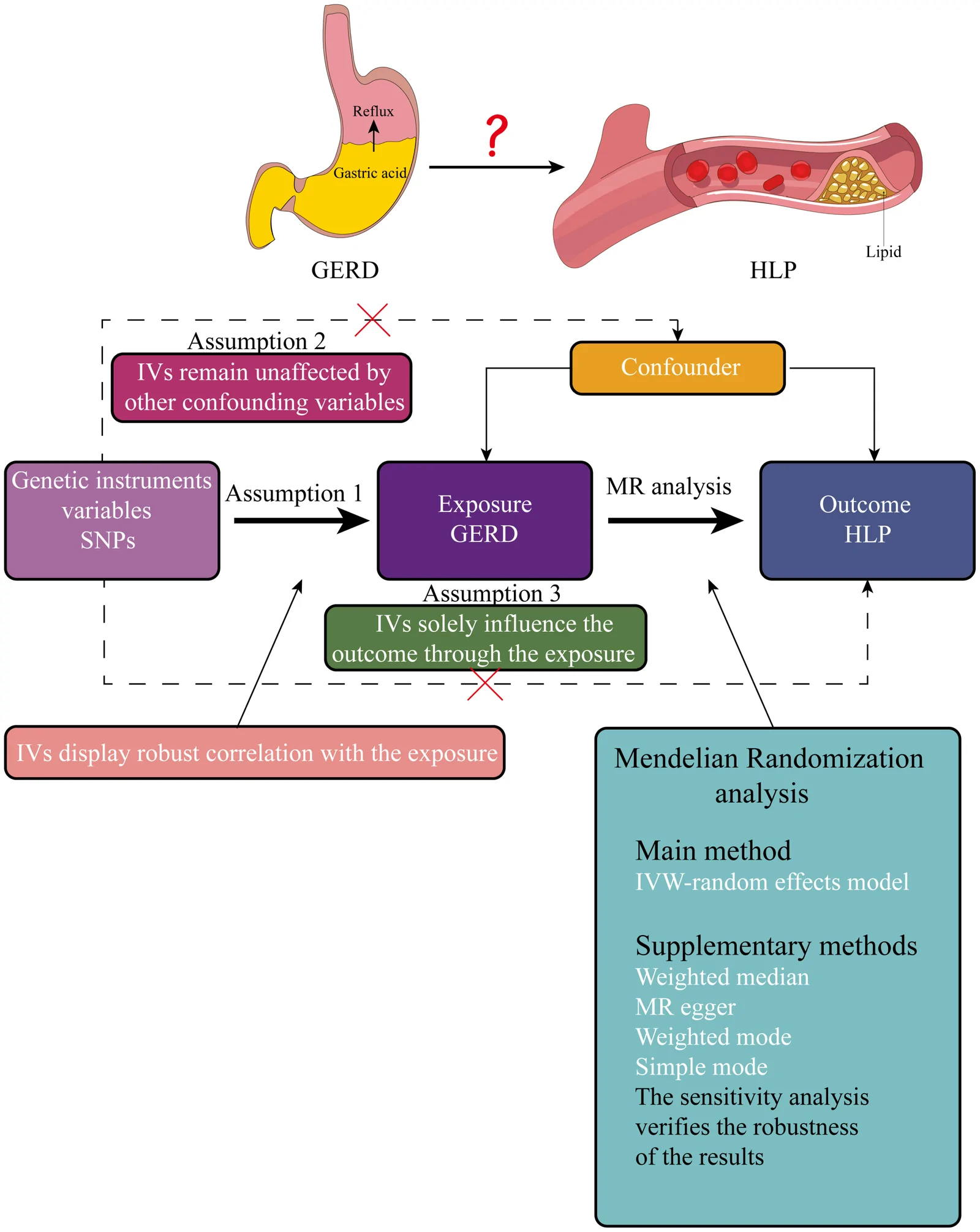
Flowchart of the MR section. GERD = gastroesophageal reflux disease, HLP = hyperlipidemia, IVW = inverse variance weighted, IVs = instrumental variables, SNPs = single nucleotide polymorphisms. Figure 2. summarizes the step-by-step MR procedure employed in this study.

This study utilized publicly available GWAS summary statistics. All data were de-identified and anonymized; therefore, ethical approval and informed consent were waived. This study followed the STROBE-MR statement.^[16]^ In this study, GERD was identified as the exposure factor and HLP as the outcome factor, respectively. The IVs were derived from the genome-wide association studies (GWAS) database. For GERD data, we extracted the most recent and extensive GWAS study from the GWAS database, encompassing 129,080 controls and 473,524 cases, culminating in a collective sample size of 602,604 individuals of European ancestry. GWAS summary data for HLP were obtained from the MRC Integrative Epidemiology Unit (MRC-IEU) Consortium, and to mitigate the potential bias from sample overlap between the exposure and outcome GWAS datasets, we performed a replication analysis using an independent HLP dataset from the FinnGen Consortium (Table 1). Access to the GWAS data utilized in the MR analysis can be attained through the MRC Integrative Epidemiology Unit (IEU) open GWAS project. The flowchart of the MR section is presented in Figure 2.

**Table 1:** Basic information on the genome-wide association studies applied in this study.

Year	Trait	Consortium	Population	Sample size	N-case	N-control	Number of SNPs
2021	GERD	PMID: 34187846	European	602,604	129,080	473,524	2,320,781
2018	HLP	MRC-IEU	European	463,010	3439	459,571	9,851,867
2021	HLP	FinnGen	European	201,794	4535	197259	16,380,389

GERD = gastroesophageal reflux disease, HLP = Hyperlipidaemia, MRC-IEU = MRC Integrative Epidemiology Unit.

### 2.5. IVs Selection

MR analysis is based on three key assumptions: IVs are strongly linked to the exposure factor, IVs are free from confounding factors, and IVs affect the outcome only through the exposure factor.^[17]^ The process of selecting IVs starts with identifying SNPs that surpass the genome-wide significance level of *P* < 5 × 10^-8^. SNPs with a linkage disequilibrium coefficient r^2^ < 0.001 and a region width > 10,000 kb were included to ensure IVs’ independence, thereby minimizing pleiotropic effects on outcomes.^[18]^ GERD-associated SNPs were derived from the GWAS data of HLP.^[19]^ SNPs directly linked to HLP were eliminated (*P* < 5 × 10^-8^).

R^2^ represents the variance proportion of the exposure factor, calculated using the formula R^2^ = 2 × (1 - EAF) × EAF × β^2^, where EAF is the effect allele frequency and β indicates the genetic impact on GERD risk.^[20]^ An F-statistic > 10 is commonly adopted to ascertain valid IVs.^[21]^ The formula F = R^2^ × (N - 2) ÷ (1 - R^2^) is used, where N represents the sample size of the exposure factor.^[22]^ Missing genotype data were not applicable in this study, as all analyses were performed on publicly available summary statistics without individual-level missingness.

### 2.6. MR analysis

Proxy SNPs were not used in the outcome data extraction (proxies = FALSE), and a fixed random seed (20240818) was set for all stochastic steps to ensure reproducibility. The inverse variance weighted (IVW) method was first employed to explore the causal relationship between GERD and HLP. Subsequently, the MR-Egger regression, Simple mode, Weighted median, and Weighted mode were employed to enhance the reliability of the results. The consistency of the outcomes of the aforementioned MR analysis methods indicates a dependable and consistent causal relationship between exposure and outcome.

### 2.7. Sensitivity analysis

Cochrane’s Q test was applied to evaluate the heterogeneity of MR analysis results, where *P* < .05 indicated the presence of heterogeneity. The MR-Egger method was applied to identify pleiotropy among the selected IVs, with *P* > .05 indicating no pleiotropy. Funnel plots were used to identify heterogeneity and evaluate symmetry; symmetrical patterns in funnel plots suggested no heterogeneity. The leave-one-out test was employed to assess the reliability of the selected SNPs. The results were considered robust due to the lack of heterogeneity and pleiotropy. Statistical analyses were executed with the “TwoSampleMR” package within R software (V4.4.1, R Foundation for Statistical Computing, Vienna, Austria).

## 3. Results

### 3.1. Identification of the shared DEGs

**Figure 3.**
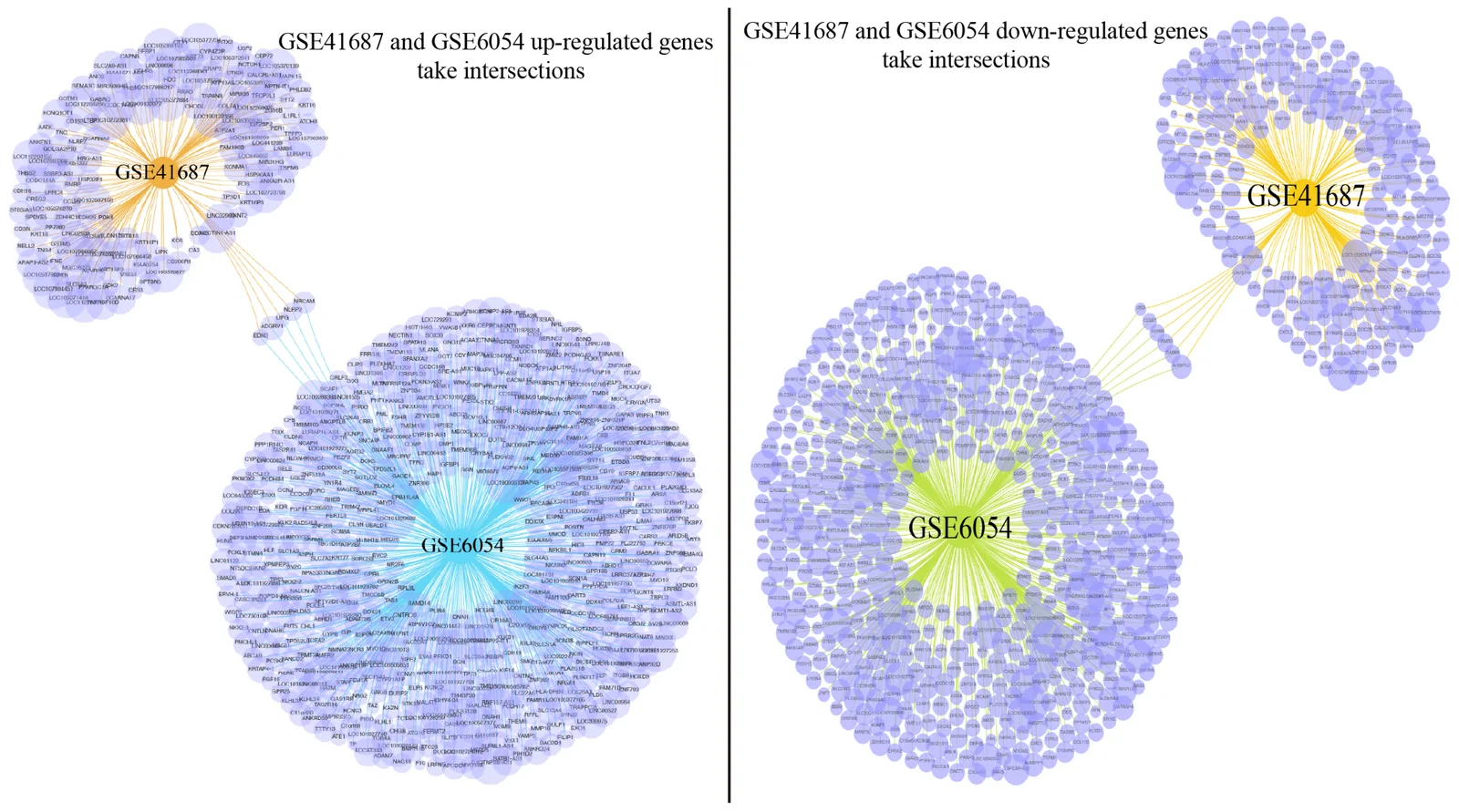
Shared DEGs (up/downregulated) between GSE41687 and GSE6054. Figure 3. displays the intersection of upregulated and downregulated genes between the 2 independent expression profiling datasets.

**Figure 4.**
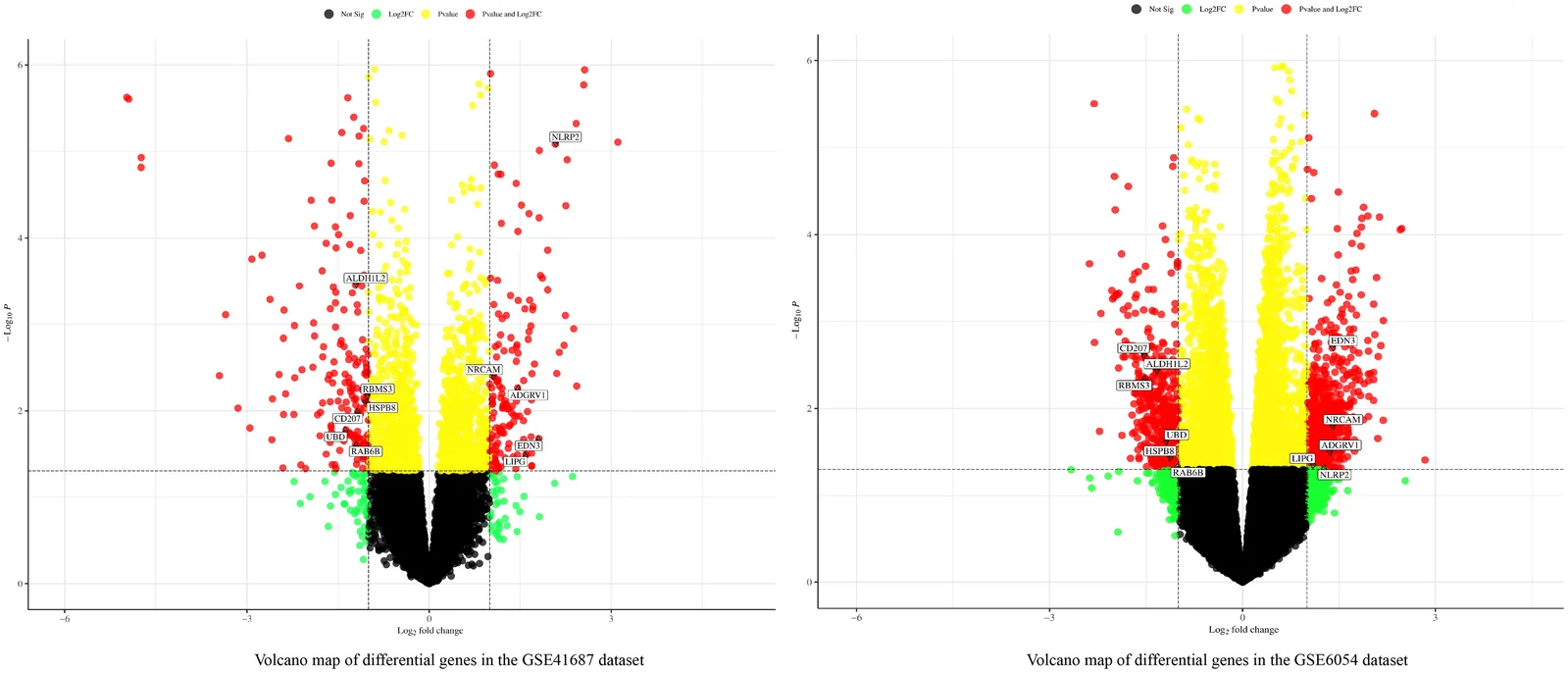
Volcano plots of gene expression in GSE41687 and GSE6054 datasets. In the visualization, black dots indicate genes without significant differential expression, green dots denote genes with *P* < .05, yellow dots signify genes with |log2FC| > 1, and red dots represent DEGs. The 11 shared genes are highlighted with annotations.

Differential analysis was executed separately on the two datasets with the online tool GEO2R from the GEO database, adhering to the criteria of *P* < .05 and |log2FC| > 1. Consequently, 332 DEGs were identified in the GSE41687 dataset, encompassing 152 upregulated genes and 180 downregulated genes (Supplemental Digital Content 1). Within the GSE6054 dataset, 1086 DEGs were obtained, comprising 595 upregulated genes and 491 downregulated genes (Supplemental Digital Content 2). Overlap of upregulated and downregulated genes from both datasets resulted in 11 shared DEGs (Fig. 3). The upregulated genes included NRCAM, EDN3, ADGRV1, NLRP2, and LIPG, whereas the downregulated genes included RBMS3, HSPB8, CD207, ALDH1L2, RAB6B, and UBD. A volcano plot was constructed for visual representation (Fig. 4).

### 3.2. GO and KEGG enrichment analyses

**Figure 5.**
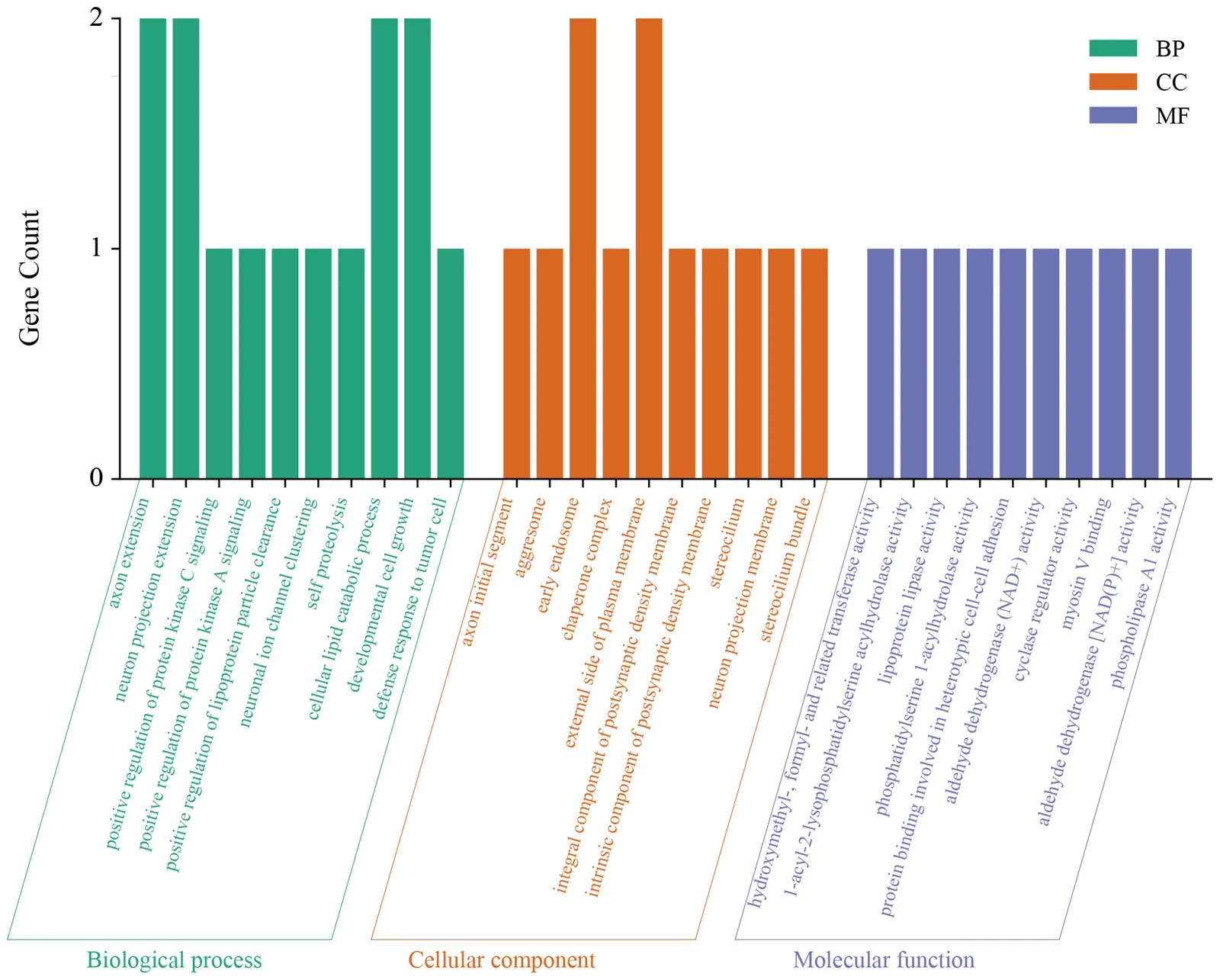
GO enrichment analysis results for shared DEGs between GSE41687 and GSE6054 datasets. Figure 5. presents the GO enrichment analysis results for the shared DEGs between the 2 datasets, highlighting the significantly enriched BP, CC, and MF that may underlie the pathogenesis of the disease.

**Figure 6.**
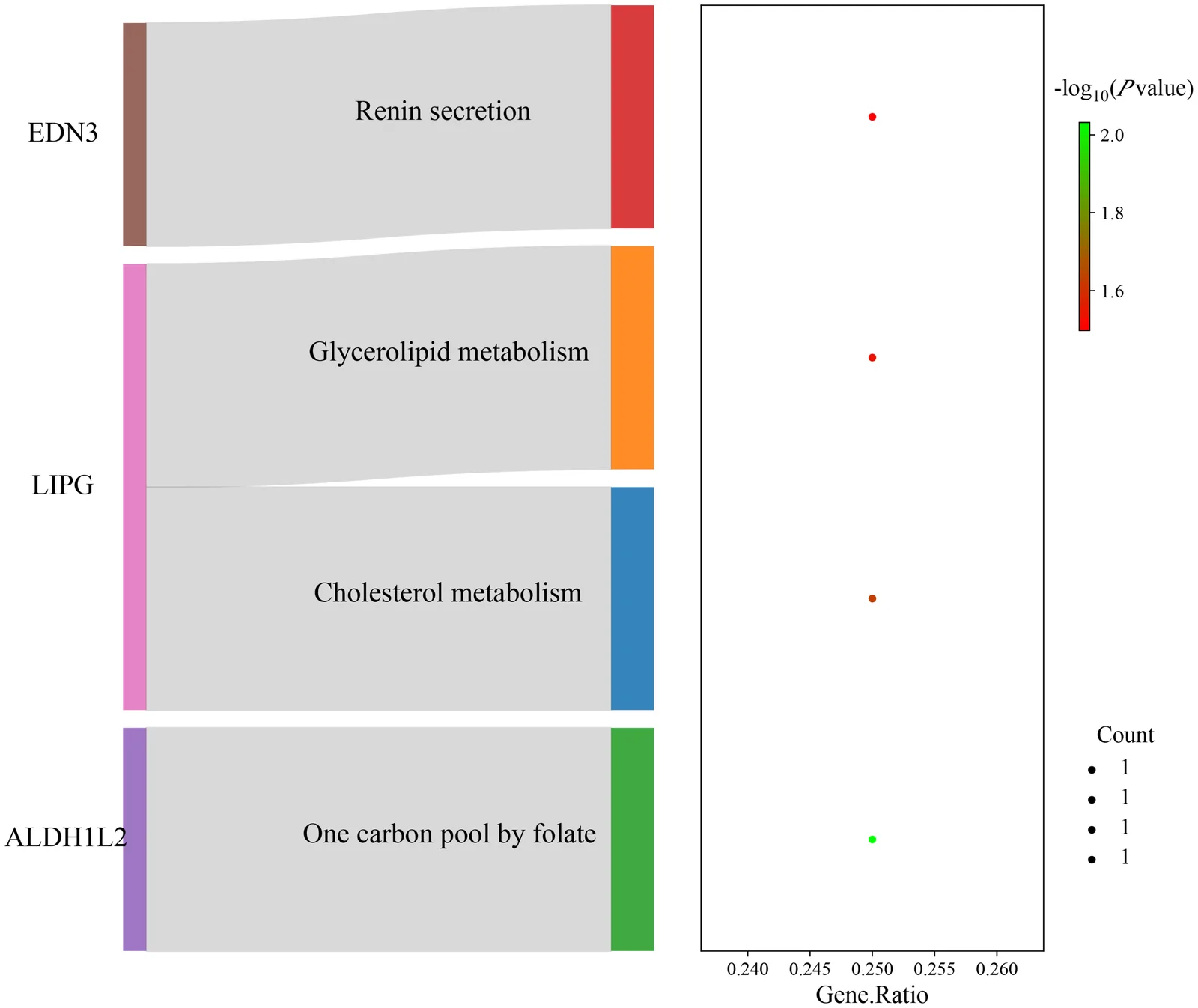
KEGG enrichment analysis results for shared DEGs between GSE41687 and GSE6054 datasets. Figure 6. presents the KEGG pathway enrichment results for the shared DEGs between the 2 datasets, identifying the significantly enriched signaling and metabolic pathways that may contribute to the molecular pathogenesis of the disease.

The shared DEGs were subjected to GO and KEGG enrichment analyses using the R package ‘clusterProfiler’ to assess the associated biological functions and pathways. This yielded 173 GO terms (Supplemental Digital Content 3). The BP category predominantly encompasses axon extension, neuron projection extension, and positive regulation of protein kinase C signaling. The CC category mainly involved the axon initial segment, aggresome, and early endosome. The MF category included hydroxymethyl-, formyl- and related transferase activity, 1-acyl-2-lysophosphatidylserine acylhydrolase activity, and lipoprotein lipase activity. KEGG analysis (Supplemental Digital Content 4) revealed four potentially enriched pathways: One carbon pool by folate, Cholesterol metabolism, Glycerolipid metabolism, and Renin secretion. Visual depictions of these results are shown in Figures 5 and 6, respectively.

### 3.3. MR analysis

**Figure 7.**
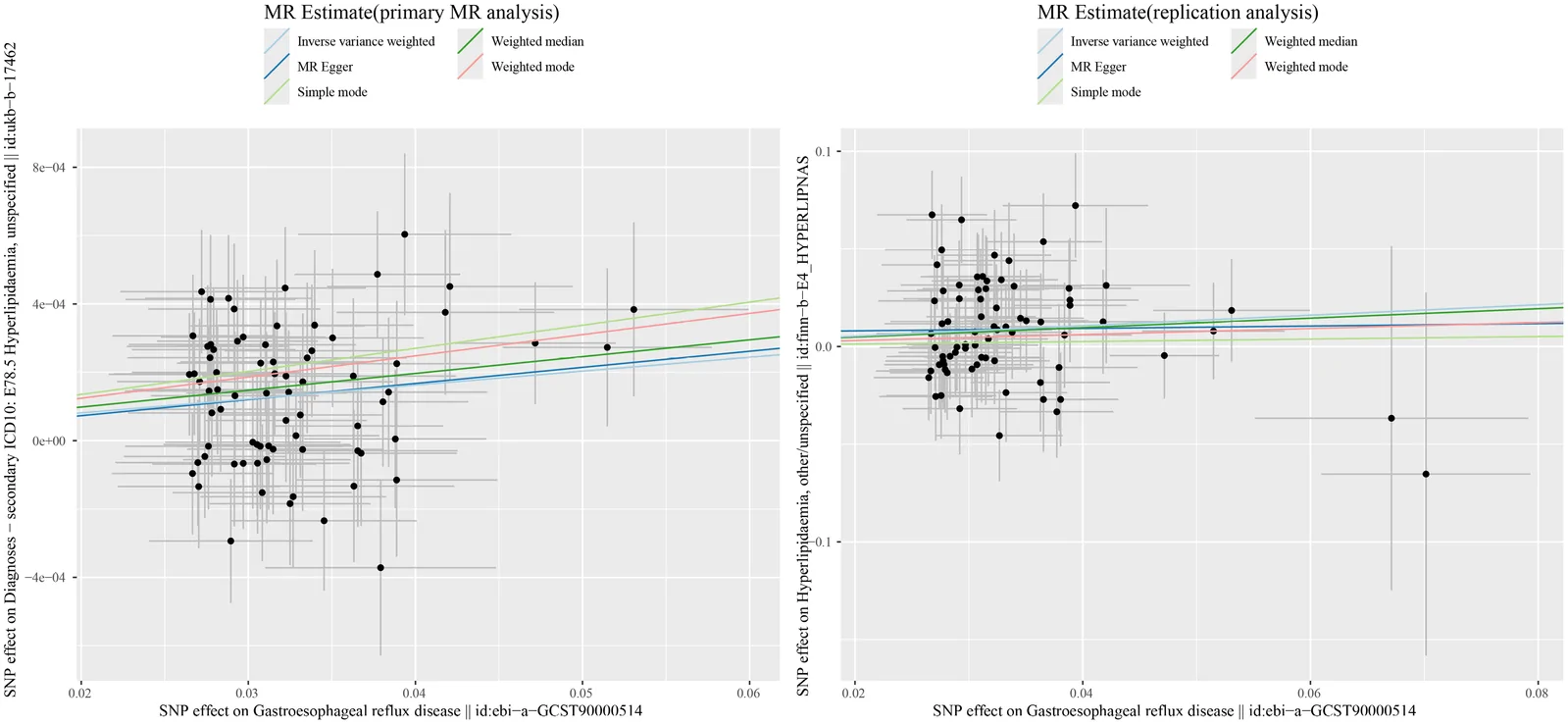
Scatterplot of MR analysis results. Each line segment represents a specific MR method, with the starting point lower than the endpoint, indicating the absence of heterogeneity.

After the exclusion of non-compliant SNPs, 75 SNPs were ultimately incorporated for both the primary MR analysis and the replication analysis; their essential details are tabulated in Supplemental Digital Content 5. In the primary MR analysis, the IVW method demonstrated a significant causal link between GERD and HLP (*P* < .001). The MR Egger (*P = *.30), Weighted median (*P* < .001), Simple mode (*P* = .01), and Weighted mode (*P* = .01) analyses supported the IVW findings. The IVW estimate remained significant in the replication analysis (*P* = .003), and although the MR Egger (*P* = .91), Weighted median (*P* = .05), Simple mode (*P* = .82), and Weighted mode (*P* = .58) did not reach statistical significance, all methods showed consistent positive effect directions. These findings reinforce the robustness of the causal relationship between GERD and HLP. MR analysis results are provided in Supplemental Digital Content 6. The scatter plot is shown in Figure 7.

### 3.4. Sensitivity analysis

**Figure 8.**
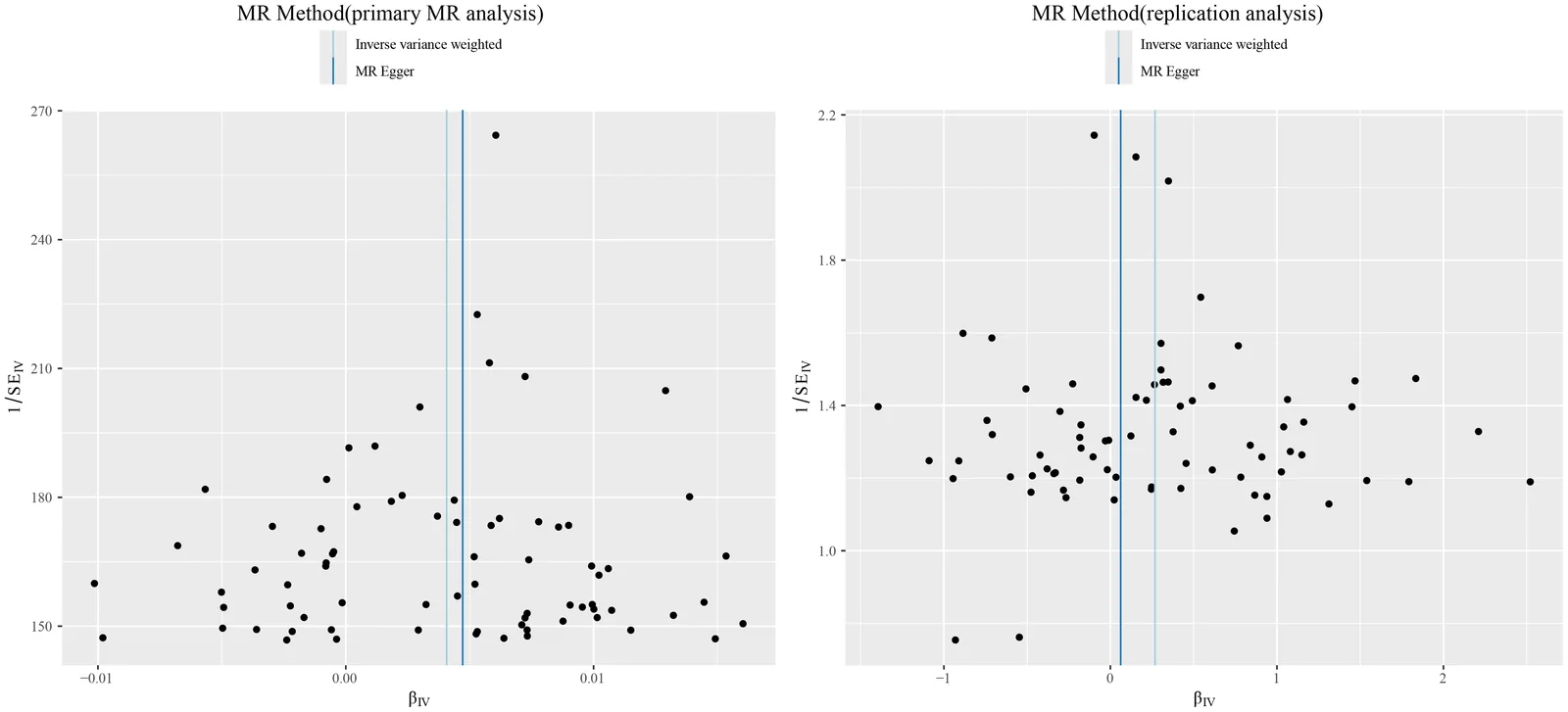
Funnel plot of MR analysis results. Each point denotes a single SNP, and the overall image exhibits approximate symmetry, ruling out the presence of heterogeneity.

**Figure 9.**
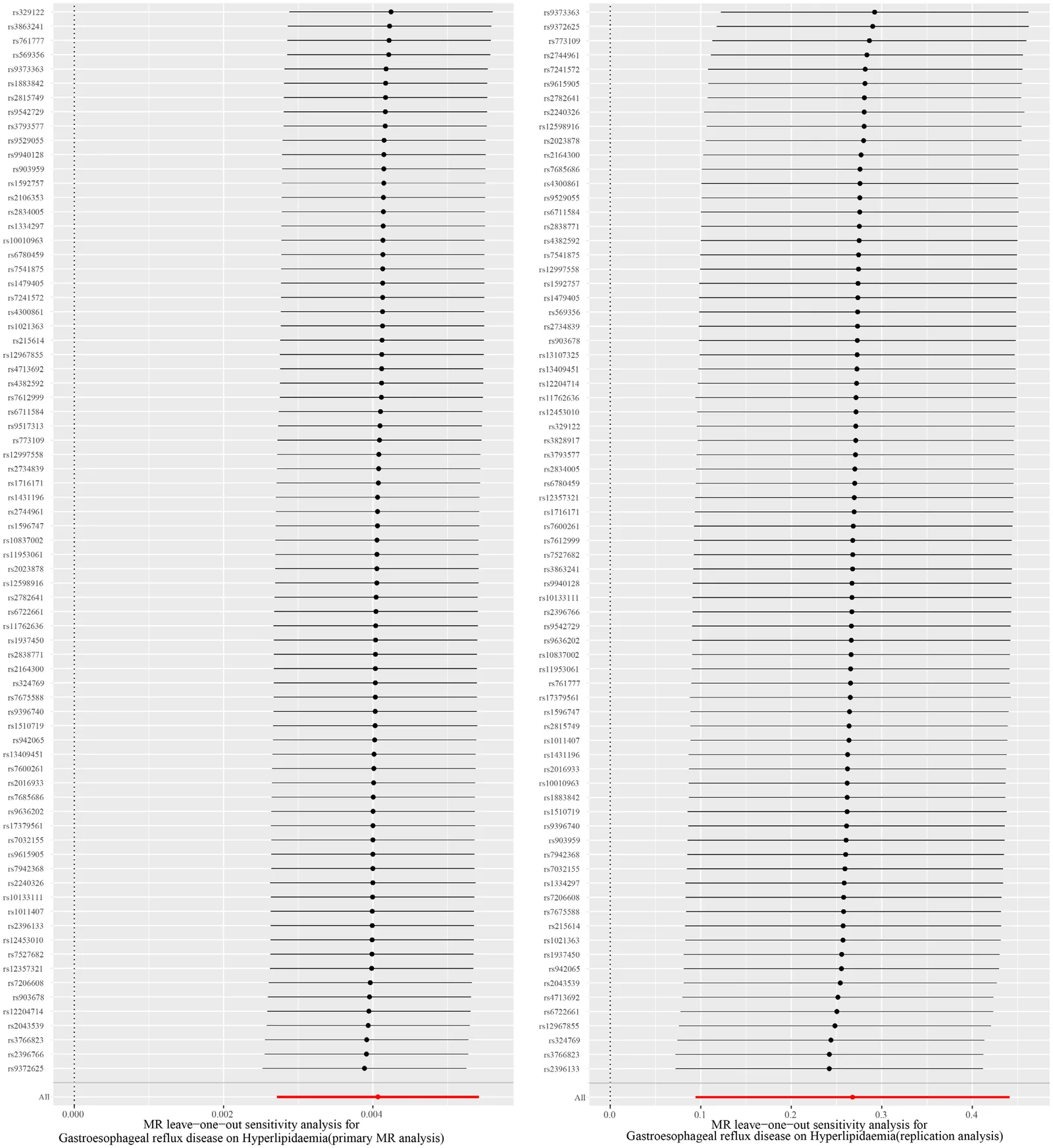
Leave-one-out test of MR analysis results. Leave-one-out test results show that even after removing any SNP, the obtained results remain unchanged, indicating that none of the SNPs are responsible for the significant findings.

Cochran’s Q test indicated no significant heterogeneity in either the primary or the replication analysis, and the Egger-intercept test showed no evidence of pleiotropy, signifying that the SNPs pertaining to GERD do not exert their influence on HLP through channels other than GERD itself (Table 2). The symmetry observed in the funnel plot (Fig. 8) further confirmed the absence of heterogeneity. The results stemming from the leave-one-out test (Fig. 9) revealed that even upon exclusion of any individual SNP, the residual outcomes remained positioned to the right of the null line. This suggests that excluding any individual SNP does not substantially affect the results, indicating the robustness of these results.

**Table 2:** Results of the heterogeneity and pleiotropy tests.

Heterogeneity test							Pleiotropy test		
Outcome	MR-Egger			IVW			MR-Egger		
	Q	Q_df	*P* *	Q	Q_df	*P* *	SE	intercept	*P* *
HLP (MRC-IEU)	73.03	73	.48	73.05	74	.51	0.0001	-2.11 × 10^-5^	.88
HLP (FinnGen)	78.04	73	.32	78.21	74	.35	0.0171	0.0068	.69

HLP = Hyperlipidaemia, IVW = Inverse variance weighted, MRC-IEU = MRC Integrative Epidemiology Unit, SE = standard error.

* *P* >.05 represents no presence of heterogeneity or pleiotropy.

## 4. Discussion

In this study, our investigation into the association between GERD and HLP, using bioinformatics and MR methods, revealed shared pathogenic mechanisms between the 2 conditions.

Metabolic syndrome (MetS), characterized by abdominal obesity, hyperglycemia, elevated TG levels, low HDL cholesterol levels, and hypertension, is a recognized risk factor for cardiovascular diseases.^[23]^ Research has suggested that MetS independently increases the risk of GERD, indicating possible lipid metabolism anomalies associated with GERD.^[24]^ The renin-angiotensin system (RAS) is composed of renin, angiotensin-converting enzyme (ACE), angiotensinogen, and is a key regulator of water, electrolytes, fluid balance, and blood pressure. Increased plasma low-density lipoprotein (LDL) cholesterol levels facilitate passive diffusion through endothelial cell junctions, resulting in its accumulation within the subendothelial layers of blood vessels. Oxidation of these LDL particles forms oxidized LDL, and connections between oxidized LDL and RAS have been established, potentially resulting in concurrent hypertension and HLP.^[25]^ Evidence indicates that the esophageal mucosa expresses RAS components. In biopsies from patients with erosive esophagitis, ACE and Angiotensin II Type 1 Receptor (AT1R) are consistently elevated compared with normal mucosa, pointing to local RAS activation as a feature of GERD. This regional RAS perturbation may extend to the systemic circulation, providing a plausible mechanistic connection between GERD and the development of HLP.^[26]^ Folic acid, also known as vitamin B9, is a vital carrier of one-carbon units that are primarily present in green leafy vegetables. Insufficient folic acid intake can disrupt the metabolism of phosphatidylcholine, thereby affecting the secretion of lipoproteins and adversely affecting hepatic lipid homeostasis.^[27]^ Therefore insufficient folic acid intake can contribute to HLP occurrence and development.

Several mechanisms may underlie the effects of GERD on HLP. Inflammatory responses in the esophageal mucosa influence GERD progression and expedite hypersensitivity to intraluminal irritation, involving cytokines like interleukin IL-1β, IL-8, and interferon INF-γ.^[28]^ Inflammation is pivotal in the development and progression of HLP, and GERD-induced inflammation potentially influences HLP via these inflammatory cytokines. Bile acid (BA) is a significant mediator of nutrient absorption, lipid secretion, and activation of nuclear receptor and G protein-coupled receptor signaling pathways. Abnormal BA metabolism can result in cholestatic liver disease, dyslipidemia, and cardiovascular diseases.^[29]^ Notably, patients with GERD exhibit increased BA concentrations in esophageal aspirates, and bile reflux exacerbates mucosal damage,^[30]^ therefore, bile reflux may disrupt the metabolic balance of BA, leading to the development and progression of HLP. Sleep disorders can negatively affect the body’s metabolism, potentially causing the development of MetS (abdominal obesity, dyslipidemia, and hypertension).^[31]^ The relationship between GERD and sleep disorders appears bidirectional, with nocturnal GERD episodes causing insomnia disorders and sleep disturbances exacerbating GERD by prolonging digestive fluid contact.^[32]^ Further research is required to validate the GERD-HLP association and identify the underlying mechanisms.

This study has several strengths. First, it initiates with a preliminary exploration of the GERD-HLP association via bioinformatics methods, followed by the application of MR analysis to delve deeper into this connection and to discuss its clinical implications. The incorporation of MR analysis effectively eradicated confounding variables and biases originating from reverse causation. Second, the use of various sensitivity analysis methods, such as the MR Egger and leave-one-out test, contributes to affirming the absence of heterogeneity and pleiotropy within the study findings, thereby amplifying the robustness of the results.

However, this study has several limitations. The study’s findings are limited in applicability to other ethnic groups due to the exclusive use of data from individuals of European ancestry. Further research is essential to ascertain the consistency of our results across diverse populations. Second, the mechanisms by which GERD influences HLP require further experimental validation. The lack of laboratory validation underscores the exploratory nature of our findings. Lastly, a potential sample overlap exists, although the F-statistic of all SNPs employed in the study (between 207 and 669) predicted minimal overlap.

## 5. Conclusions

This study concluded that GERD and HLP share pathogenic genes and mechanisms, identifying GERD as a risk factor for HLP. Therapeutic strategies for GERD may also be effective in preventing or improving HLP. These findings provide valuable insights into the clinical management of both conditions and enhance preventive and therapeutic strategies.

## Acknowledgments

We wish to express our appreciation to TopEdit (www.topedits.com) for their assistance in the preparation of this manuscript. The authors acknowledge the MRC-IEU Consortium and GEO databases for providing high-quality data for the researchers. We also thank Shanghai NewCore Biotechnology Co., Ltd. (https://www.bioinformatics.com.cn) and OmicShare (https://www.omicshare.com/tools/) for providing visual support.

## Author contributions

**Investigation:** Yongming Sun, Wodele Kang.

**Methodology:** Yongming Sun, Dawei Cheng.

**Visualization:** Yongming Sun.

**Writing – original draft:** Yongming Sun, Xiaodong Xing.

**Writing – review & editing:** Yongming Sun, Wenyan Wang, Jingbo Zhai.

**Project administration:** Jingbo Zhai.

**Supervision:** Jingbo Zhai.












